# Post‐9/11 Veterans Military‐to‐Civilian Transitions: Predictors of Mental Health Symptoms Over the First 3 Years

**DOI:** 10.1002/jclp.70038

**Published:** 2025-08-18

**Authors:** Mary M. Mitchell, Keith R. Aronson, Daniel F. Perkins

**Affiliations:** ^1^ Clearinghouse for Military Family Readiness Pennsylvania State University University Park Pennsylvania USA

**Keywords:** adverse childhood events, anxiety, depression, military deployments, military‐to‐civilian transitions, veterans

## Abstract

**Objective:**

Many post‐9/11 veterans struggle with psychological symptoms as they transition to civilian life. Adverse childhood experiences, combat exposure, and deployment characteristics are factors associated with symptoms. This study examined changes in the predictive power of these factors over the first 3 years of the military‐to‐civilian transition among post‐9/11 veterans.

**Methods:**

This was a longitudinal survey study in which six waves of data were collected over 3 years.

**Results:**

The associations between combat, ACEs, deployment characteristics, and psychological symptoms were complex, not static, not always linear, and differed between male and female veterans. The number of deployments was associated with fewer psychological symptoms at baseline for both genders. For males, longer deployments at baseline predicted worse mental health, while more deployments were associated with improving mental health over time.

**Conclusion:**

Temporal explorations of veteran mental health are needed to gain insights into how and when psychological problems develop and change over time. Veterans need a robust support network to prevent mental health problems as they transition to civilian life.

## 1 Introduction

While most post‐9/11 veterans successfully navigate the military‐to‐civilian transition (MCT), a substantial minority struggle with this process (Karre et al. 2024; Park et al. 2021). More than 44% of post‐9/11 veterans report challenges with transitioning from active duty to civilian life, and 27% report their transition was somewhat or very difficult (Morin 2011). There is also evidence that the first year of the transition to civilian life can be particularly stressful for veterans (Karre et al. 2024).

Transitions to civilian life require veterans to re‐engage with family and friends, find employment, settle into a new community, and possibly pursue additional education among other adjustments (Ahern et al. 2015). Veterans must also contend with losing their military identity, regimentation, and close military friends and colleagues (Pedlar et al. 2019). Post‐9/11 veterans further deal with physical and mental health challenges, and this group of veterans has the most service‐connected disabilities and the highest disability ratings in the nation's history (US Bureau of Labor Statistics 2020). Understanding how MTCs unfold over time has important implications when choosing public‐health approaches that best enhance care for post‐9/11 veterans (Bokhour et al. 2020; Karre et al. 2024).

There are several theoretical models posited to explain MTCs, although none has been widely accepted. Life course theory (Pedlar et al. 2019) suggests that the quality or successfulness of MTCs is contingent upon pre‐military service (e.g., exposure to adverse childhood events, level of education), during military service (e.g., combat exposure, traumatic experiences), and post‐military service (e.g., access to healthcare and social support) factors. These factors can act as resilience building or risk inducing phenomena. In general, the life course theory suggests that exposure to risk factors before, during, and after military service lead to poorer MCT outcomes, while exposure to resilience building factors lead to better MCTs.

### 1.1 Effects of Adverse Childhood Experiences (ACE), Deployment, and Combat on Mental Health

#### 1.1.1 Pre‐Military Factors

One pre‐military service factors related to MCTs is exposure to ACEs. ACEs include being a survivor of physical, psychological, or sexual abuse, as well as experiencing neglect. Other family circumstances such as having an incarcerated parent or substance abuse are also adverse experiences. Veterans are more likely to experience ACEs than civilians. While approximately half of the general population in the United States reports having experienced at least one ACE, estimates for veterans tend to be higher, although they are variable. A study of veterans using outpatient services at the VA found 85% reported experiencing at least one ACE (Laird and Alexander 2019). Data from the 2010 Behavioral Risk Factor Surveillance System found approximately 70% of veterans from all wars reported at least one exposure (Blosnich et al. 2014). Approximately 50% of females entered the military having experienced physical or sexual abuse (Institute of Medicine 2013). In a study of post‐9/11 community dwelling veterans, approximately 59% of female and 40% of male veterans reported experiencing at least one ACE (Aronson et al. 2020).

Exposure to ACEs has deleterious consequences. Many studies of both civilians and veterans have shown that ACEs are associated with numerous problematic health outcomes. For example, exposure to ACEs is a significant risk factor for the development of adult mental health problems and premature mortality (Anda et al. 2006). ACEs are also associated with mental health disorders among veterans, including depression, PTSD, and substance abuse (Institute of Medicine 2013).

#### 1.1.2 During Military Factors

Physical and mental health can be affected by military service. Mental health concerns, such as depression, anxiety, and trauma‐related disorders, are not uncommon among post‐9/11 veterans (Inoue et al. 2021). The impact of mental health problems can last for years and influence how well veterans and their families adapt to civilian life (Cramm et al. 2020). Due to the mental health challenges that impact veterans during their MCTs, some scholars suggest that support and programs should focus on psychological concerns instead of centering on vocational issues (Joseph et al. 2023). In addition, a sizeable number of post‐9/11 veterans report that obtaining mental healthcare is a pressing problem due to barriers to care such as wait times, geographic isolation, and navigating the DoD and VA systems upon leaving the military (Derefinko et al. 2019).

### 1.2 Post‐Military Factors

Deployment, even in the absence of combat exposure, increases the risk of veterans developing mental health disorders, including anxiety, depression, substance use, suicidality, and post‐traumatic stress disorder (PTSD) (Institute of Medicine 2010; Sayer et al. 2014. A common non‐combat stressor, long deployment length, was rated as highly or very highly distressing by 52% of warfighters returning from deployment during Operation Iraqi Freedom (US Army Surgeon General 2005). In addition, multiple deployments place service members at risk for developing PTSD; however, less is known about the risks of developing other mental health disorders (Institute of Medicine 2013). Exposure to combat includes engaging in occurrences during military confrontations and observing the aftermath of combat (Gaylord 2006). Veterans exposed to combat are at risk for developing PTSD, depression, anxiety, substance abuse, and suicidality and for having comorbid conditions (Galovski and Lyons 2004; Milanak et al. 2013; Norman et al. 2018; Sher et al. 2012). Combat‐deployed post‐9/11 service members were at greater risk for developing mental health disorders compared to those who were deployed but did not experience combat (Campbell et al. 2021). Combat exposure also creates a risk factor for a service member to experience moral injury, which can be associated with feelings of guilt, sadness, and self‐blame (Chesnut et al. 2020). Finally, after serving, veterans make MCTs, a process that is often stressful and challenging (Morin 2011).

### 1.3 The Current Study

The current investigation explored the longitudinal trends in self‐reported psychological symptoms among post‐9/11 veterans within approximately the first 3‐years following their separation from military service. Using life course theory of MCTs, the study explored the main effects of exposure to ACEs (i.e., premilitary service deployment characteristics) and during military service experiences including the number of deployments, length of the longest deployment, and combat exposure on mental health problem trajectories over the first 3‐years of post‐9/11 veteran MCTs. Furthermore, the study included tests of the effects of adding interaction terms between pairs of predictors, including between deployment variables and ACEs and between deployment variables and combat exposure. Longitudinal trends and predictor effects were examined separately for male and female veterans as they have divergent incidence of both exposure to ACEs (females have higher exposure rates) and psychological symptoms (females report more symptoms). The study hypothesized that (a) psychological symptoms would decline (i.e., improving mental health) over time; (b) combat exposure, deployments (both number and length), and exposure to ACEs would be associated with higher psychological symptoms at baseline; and (c) exposure to combat, deployments, and ACEs would also be associated with a slower rate of decline in psychological symptoms over time such that the symptoms would be more likely to persist rather than resolve.

## 2 Materials and Methods

### 2.1 Participants

All veterans who separated from active duty service no more than 3 months before August 9, 2016, to September 20, 2016, from any of the Service branches were invited to participate in The Veterans Metric Initiative (TVMI) study, which has been described in previously published work (Perkins et al. 2020; Vogt et al. 2018). Twenty percent (*n* = 9566) of the invited veterans completed the baseline survey. At baseline, most participants were male (*n* = 7823; 81.8%) and White (*n* = 6826 (71.4%), African American/Black (*n* = 1066; 11.2%), and approximately 16% were Hispanic. The mean age of the participants was 34.5 years with a standard deviation of 9.5 years (for full sample demographic characteristics, see Vogt et al. 2018). Mean length of deployments among Veterans in this sample was 6.47 months (SD = 5.33) with a range of 0‐60 months. On average, they were deployed 1.86 times (SD = 2.09) with a range of 0−10. Slightly more than half (53.4%) reported one or more combat exposures.

### 2.2 Measures

#### 2.2.1 Psychological Symptoms

Psychological symptoms, the main outcome variable in this study, were measured using the 4‐item version of the Patient Health Questionnaire (PHQ‐4) (Kroenke et al. 2009). The PHQ‐4 asks respondents to indicate how often (not at all = 0, several days = 1, more than half the days = 2, and nearly every day = 3) over the prior 2 weeks they were bothered by symptoms of anxiety (e.g., not being able to stop or control worrying) and depression (e.g., feeling down, depressed, hopeless). Scale scores were summed. At each time point, Cronbach's alpha for each summed scale score was 0.89.

#### 2.2.2 Exposure to ACEs

ACEs were measured using a 7‐item questionnaire modified from the ACEs Study (Felitti et al. 1998) that was used in a study that examined the impact of ACEs on the risk of developing post‐deployment PTSD among a large sample of Marines. (LeardMann et al. 2010) Participants were asked to retrospectively indicate (yes/no) whether they had experienced any of seven ACEs (e.g., physical neglect, physical abuse) before the age of 17. Items were summed to create the total number of adverse experiences. The ACEs questionnaire was included in the Wave 5 survey of the TVMI study (approximately 24−27 months after separating from the military).

#### 2.2.3 Combat Exposure

Combat exposure and exposure to the aftermath of combat were measured with a 9‐item scale derived from the Deployment Risk and Resilience Inventory (King et al. 2003; Vogt et al. 2013). Respondents indicated how often they encountered combat‐related events, such as firing a weapon or encountering improvised explosive devices. They also reported on witnessing the aftermath of battle such as seeing civilians after they had been severely wounded or disfigured or observing the destruction associated with bombings. Response options included 0 (*never*), 1 (*once or twice*), 2 (*several times*), and 3 (*many times*). Due to non‐normal distribution, response options were recoded into a dichotomized variable of 1 (*at least once*) and 0 (*never*).

### 2.3 Procedures

Institutional Review Board approval was obtained. In brief, each post‐9/11 veteran was identified from the Veterans Affairs/Department of Defense Identity Repository (VADIR) benefits database. Each veteran who separated from the military within the 90 days before August 9, 2016, to September 20, 2016, was invited to participate in a longitudinal research study that intended to examine their transition experience from military to civilian life. The invitations included a pre‐alert postcard, a notification letter containing a $5 pre‐incentive, and two postcard reminders. In addition to the $5 cash incentive, veterans who completed the survey received a $20 Amazon.com gift card. The online survey took approximately 45 min to complete. Five additional surveys were administered at approximately 6‐month intervals. A detailed explanation of the procedures used has been published (Vogt et al. 2018).

### 2.4 Data‐Analytic Plan

Frequencies and means were generated for study variables, including the summed scores of the mental health problems items at each time point and the five time‐invariant predictors, which included the number of times deployed and the longest length of deployment; ACEs scores, which were comprised of two dummy variables (endorsed 1−2 ACEs and endorsed 3 or more ACEs, compared to the reference category of endorsing zero ACEs); and combat exposure.

**Figure 1:**
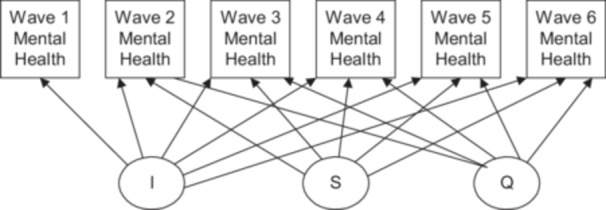
Measurement model with six waves of mental health measures as indicators for the intercept, slope and quadratic latent factors in latent growth curve analyses (*N* = 9566).

Next, an unconditional latent growth curve (Curran 2000) model was fit to the data using the mental health problem items summed at each time point (see Figure 1 for the measurement model). The maximum likelihood robust estimator that employs Full Information Maximum Likelihood for outcomes with skewed distributions was used. Based on the Comparative Fit Index (CFI) value increasing by 0.012 between the model with intercept and slope versus the model with intercept, slope, and quadratic term, the latter was accepted as preferable. The variances for all three growth factors were significant at *p* < 0.001, which provided additional evidence for retaining all three latent factors in the model.

Conditional latent growth models were fit separately for women (*n* = 1743) and men (*n* = 7822). In the main effects model, the latent factors were regressed on five predictors, which included longest deployment, number of deployments, ACEs scores represented by two dummy variables, and combat exposure. Six interaction terms were included with the main effect terms for the interaction effects model. Interaction terms included number of times deployed x ACEs score of 1−2, number of times deployed x ACEs score of 3 or more, length of deployment x ACEs score of 1−2, length of deployment x ACEs score of 3 or more, number of times deployed x combat exposure, and length of deployment x combat exposure. Significant interaction regression coefficients were explored by splitting the sample by the dichotomous (e.g., combat exposure) or trichotomous (ACEs dummy variables of 0, 1−2, and 3+) variables and examining the relationship between the continuous variable in the interaction term (e.g., number of times deployed or length of deployment) and the latent factor outcome.

Missing data due to sample attrition were handled using the auxiliary variable function in Mplus v.8 (Muthen and Muthen 2017). Variables that were correlated with missing values at the baseline survey administration (Vogt et al. 2018) were included in the auxiliary variable function to make the data set better fit the missing at random (MAR) assumption. Achieving MAR increases power and reduces bias. The auxiliary variables included age, education, pay grade, race, marital status, and National Guard or Reserve status. In addition, variables that measured the presence of a geo‐codable address and medical insurance were included.

A weight was applied to adjust the results based on the sample respondents' proportions versus the general population characteristics of post‐9/11 veterans from 2016. The sample weights were calculated based on the gender, pay grade, and Service branch proportions of veterans in the larger population of post‐9/11 veterans from which the sample was drawn (Vogt et al. 2018).

## 3 Results

### 3.1 Psychological Symptoms, Exposure to ACEs, and Combat Exposure

Means over the six waves of psychological symptom scores ranged from 2.86 to 3.02, and standard deviations ranged from 3.50 to 3.67. The total scale summed scores ranged from 0 to 15. Cronbach's alpha was 0.89 for each of the summed scales. On average, female veterans reported being exposed to more ACEs (*M* = 1.86, SD = 2.14) than men (*M* = 1.09, SD = 1.76). On the other hand, female veterans reported significantly less exposure to combat (*M* = 1.95, SD = 3.92) than their male veteran peers (5.74, SD = 7.56). Table 1 includes descriptive statistics and correlations among variables.

**Table 1:** Descriptive statistics and correlations.

Variable	*N*	Mean (SD) or percent	Skew	Kurtosis	Range
No. of times deployed	9547	1.86 (2.09)	1.67	3.09	0−10
Length of longest deployment	9543	6.47 (5.33)	0.76	3.91	0−60
ACES category	5877				
0		56.9%	NA	NA	NA
1−2		22.5%	NA	NA	NA
3+		20.5%	NA	NA	NA
Combat Exposure (One or more vs. None)	9553	46.6%	NA	NA	NA

Variables	No. of times deployed	Length of longest deployment	ACES	Combat exposure
No. of times deployed	1.00			
Length of longest deployment	**0.50**	1.00		
ACES	0.01	**0.06**	1.00	
Combat exposure	**0.68**	**0.66**	**0.04**	1.00

*Note:* Bolded coefficients are significant at *p* < 0.05 or below.

Significant effects existed for ACEs and combat exposure for both males and females. Female veterans (32.1%) were significantly more likely to report exposure to three or more ACEs compared to male veterans (18.0%). Male post‐9/11 veterans (60.4%) were significantly more likely than their female peers (41.2%) to report that they had never been exposed to ACEs (*χ* ^2^ = 153.0, *p* < 0.001). The odds of having been exposed to one or more combat experiences was 2.64 times higher for male veterans compared to female veterans (Odds Ratio [OR] = 2.64, 95% Confidence Interval [CI] = [2.37, 2.94] with a *χ* ^2^ = 320.23, *p* < 0.001).

### 3.2 Associations Among Deployment Characteristics, ACEs, and Psychological Symptoms

A multiple‐group latent growth curve model was fit for men and women separately. The fit of the main effects model was excellent with the CFI = 0.998, the Tucker‐Lewis Index (TLI) = 0.995, Standardized Root Mean Square Residual (SRMR) = 0.008, and the Root Mean Square Error of Approximation (RMSEA) = 0.015 with a 90% Confidence Interval (90% CI) of [0.011, 0.019] (Hu and Bentler 1999).

**Figure 2:**
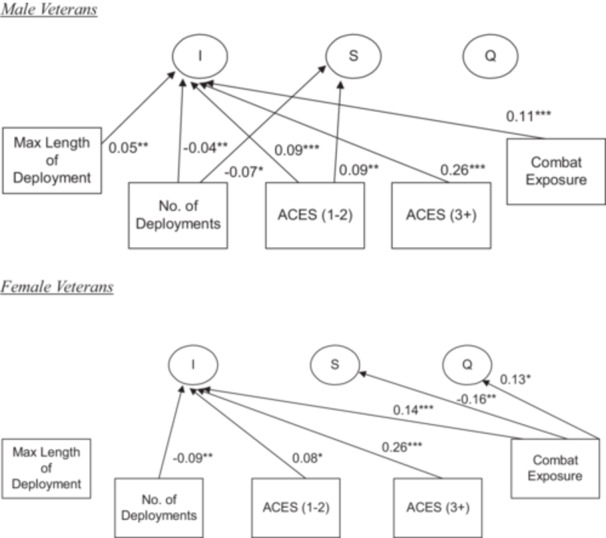
Structural portions of a multiple‐group latent growth curve model of psychological symptoms for male (*n* = 7822) and female veterans (*n* = 1743). Coefficients are standardized Beta (β) weights. *p < 0.05, **p < 0.01, ***p < 0.001. *Note:* Model fit statistics for the multiple‐group main effects model: Comparative Fit Index (CFI) = 0.998, the Tucker‐Lewis Index (TLI) = 0.995, Standardized Root Mean Square Residual (SRMR) = 0.008, and the Root Mean Square Error of Approximation (RMSEA) = 0.015 with a 90% Confidence Interval (90% CI) of [0.011, 0.019].

For men, in line with expectations, the maximum length of deployment was associated with more psychological symptoms at baseline (unstandardized *b* = 0.029, standard error [SE = 0.011], *p* = 0.008, *β* = 0.049) (Table 2, Figure 2 top). Contrary to expectation, there was an inverse relationship between the number of deployments and psychological symptoms at baseline (unstandardized *b* = −0.064 [SE = 0.023], *p* = 0.006, *β* = −0.040). Thus, male veterans who reported more deployments reported fewer psychological symptoms. Males with more deployments had a steeper decline in the slope of psychological symptom reports over time (unstandardized *b* = −0.033 [SE = 0.014], *p* = 0.016, *β* = −0.065). There was a positive association between combat exposures and psychological symptoms at baseline (unstandardized *b* = 0.689 [SE = 0.118], *p* < 0.001, *β* = 0.109). As expected, compared to men who were not exposed to ACEs, men exposed to 1−2 ACEs reported more mental health problems at baseline (unstandardized *b* = 0.718 [SE = 0.137], *p* < 0.001, *β* = 0.093) as did men who reported exposure to 3 or more ACEs (unstandardized *b* = 2.128 [SE = 0.167], *p* < 0.001, *β* = 0.257). Moreover, veterans who reported three or more ACEs reported significantly more psychological symptoms at baseline than those who reported 1−2 ACEs.

**Table 2:** Predictors of psychological symptoms for post‐9/11 veterans (*N* = 9566).

	Male			Female		
Predictors	Intercept	Slope	Quadratic	Intercept	Slope	Quadratic
Length of deployment	**0.029 (0.011)** **	0.006 (0.006)	−0.001 (0.001)	0.006 (0.025)	0.027 (0.015)	−0.004 (0.003)
No. times deployed	**−0.064 (0.023)** **	**−0.033 (0.014)** *	0.004 (0.003)	**−0.260 (0.080)** **	−0.027 (0.046)	0.007 (0.009)
Combat exposure	**0.689 (0.118)** ***	−0.079 (0.068)	0.009 (0.013)	**1.091 (0.257)** ***	**−0.501 (0.163)** **	**0.079 (0.033)** *
ACES score						
0	Ref.	Ref.	Ref.	Ref.	Ref.	Ref.
1−2	**0.718 (0.137)** ***	**0.226 (0.078)** **	−0.027 (0.015)	**0.668 (0.293)** *	0.111 (0.173)	−0.011 (0.034)
3+	**2.128 (0.167)** ***	0.027 (0.096)	0.007 (0.018)	**2.017 (0.298)** ***	−0.005 (0.166)	0.016 (0.032)

	Male			Female		
	Intercept	Slope	Quadratic	Intercept	Slope	Quadratic
Length of deployment	0.015 (0.019)	0.003 (0.011)	0.000 (0.002)	−0.011 (0.045)	0.035 (0.027)	−0.005 (0.005)
No. times deployed	−0.086 (0.054)	−0.034 (0.033)	0.005 (0.006)	**−0.306 (0.141)** *	−0.048 (0.089)	0.013 (0.016)
Combat exposure	−0.141 (0.211)	−0.019 (0.114)	0.002 (0.022)	−0.826 (0.506)	0.128 (0.294)	−0.007 (0.061)
ACES score						
0	Ref.	Ref.	Ref.	Ref.	Ref.	Ref.
1−2	**1.246 (0.226)** ***	0.012 (0.133)	0.025 (0.026)	0.725 (0.436)	0.226 (0.244)	−0.031 (0.046)
3+	**2.523 (0.272)** ***	0.089 (0.168)	−0.006 (0.032)	**2.307 (0.442)** ***	−0.181 (0.241)	0.052 (0.046)
*Interactions*						
No. times deployed x ACES 1−2	0.092 (0.166)	−0.017 (0.040)	0.001 (0.008)	−0.037 (0.246)	−0.134 (0.140)	0.032 (0.028)
No. times deployed x ACES 3+	**0.172 (0.086)** *	**−0.112 (0.052)** *	0.014 (0.010)	−0.152 (0.228)	−0.071 (0.113)	0.001 (0.021)
Length of deployment x ACES 1−2	**−0.107 (0.029)** ***	**0.037 (0.017)** *	**−0.008 (0.003)** *	−0.022 (0.070)	0.003 (0.044)	−0.002 (0.009)
Length of Deployment x ACES 3+	**−0.112 (0.035)** **	0.021 (0.022)	−0.002 (0.004)	−0.043 (0.066)	0.055 (0.037)	−0.008 (0.007)
No. times deployed x combat exposure	−0.005 (0.057)	0.026 (0.036)	−0.004 (0.007)	0.305 (0.167)	0.055 (0.108)	−0.013 (0.020)
Length of deployment x combat exposure	**0.124 (0.025)** ***	−0.014 (0.014)	0.002 (0.003)	**0.185 (0.054)** **	**−0.087 (0.035)** *	0.013 (0.007)

*Note:* Bolded unstandardized estimates indicate significance at *p* < 0.05.

* *p* < 0.05;

** *p* < 0.01;

*** *p* < 0.001.

Contrary to expectation, women who were deployed more times reported fewer psychological symptoms at baseline (unstandardized *b* = −0.260 [SE = 0.080], *p* = 0.001, *β* = −0.092), while there were no significant main effects of maximum length of deployment (Table 2, Figure 2 bottom). Female veterans who had combat exposure reported more mental health problems at baseline (unstandardized *b* = 1.091 [SE = 0.257], *p* < 0.001, *β* = 0.138) compared to those with no combat exposure. From Wave 1 to Wave 2, female veterans with combat exposure had a steeper negative slope, which indicates declining mental health symptoms between Waves 1 and 2 compared to those with no combat exposure (unstandardized *b* = −0.501 [SE = 0.163], *p* = 0.002, *β* = −0.156). Female veterans also had a significantly positive quadratic effect with greater combat exposure (unstandardized *b* = 0.079 [SE = 0.033], *p* = 0.016, *β* = 0.131). Compared to women reporting no ACEs, women with a score of 1−2 on the ACEs reported significantly greater mental health problems (unstandardized *b* = 0.668 [SE = 0.293], *p* = 0.023, *β* = 0.082) as did women who scored 3 or more on the ACEs (unstandardized *b* = 2.017 [SE = 0.298], *p* < 0.001, *β* = 0.262). Female veterans who scored 3 or more on the ACEs reported significantly greater psychological symptoms than women who scored in the 1−2 range on the ACEs.

Testing the equality of regression paths between models for men and women in the main effects and interaction models yielded three significant results. The number of times deployed was negatively associated with mental health symptoms for both men (unstandardized *b* = −0.064) and women (unstandardized *b* = −0.260). This association was significantly greater for females. The regressions for the slope and quadratic factors on combat exposure were significant for women but not men. Constraining each of these pairs of paths to be equal resulted in significant difference tests with a steeper declining slope for women who experienced combat exposure and a greater positive relationship between combat exposure and the quadratic factor for women. Thus, women who had combat exposure had a steeper decline between Waves 1 and 2 in psychological symptoms than male veterans. Female veterans with combat exposure also had a *U*‐shape upturn in symptoms over time.

### 3.3 Analyses of Interaction Effects Among Male Veterans

A series of six two‐way interactions were added to the main effects model to create a second model with interactions between the number of times deployed x ACEs (1−2 ACEs vs. 0 ACEs and 3+ ACEs vs. 0 ACEs), length of deployment x ACEs (1−2 ACEs vs. 0 ACEs and 3+ ACEs vs. 0 ACEs), number of times deployed x combat exposure, and maximum length of deployment x combat exposure. Seven of the tested interactions were significant for men, and two were significant for women.

For men, regressing the intercept and slope on the interaction of the number of times deployed x ACEs (3+) yielded significant results (unstandardized *b* = 0.172 [SE = 0.086], *p* = 0.045) and (unstandardized *b* = −0.112 [SE = 0.052], *p* = 0.032), respectively. Splitting the sample by ACEs scores showed that men with an ACEs score of 0 had a significant negative relationship between the number of times deployed and psychological symptoms (unstandardized *b* = −0.167 [SE = 0.034], *p* < 0.001). In contrast, no such significant relationship existed for men who scored 3 or more on the ACEs scale. Therefore, this significant association indicates that men who scored a 0 on the ACEs scale had fewer mental health problems at baseline compared to men with three or more ACEs. For men who scored 3 or more on the ACEs scale, a significant relationship existed between the number of times deployed and a decrease in psychological symptoms over time (unstandardized *b* = −0.122 [SE = 0.045], *p* = 0.006); however, no such significant relationship existed for men who scored 0 on the ACEs scale.

The effect of the interaction between the length of deployment and scoring a 3 or higher on the ACEs scale for men was significantly associated with baseline psychological symptoms scores (unstandardized *b* = −0.112 [SE = 0.035], *p* = 0.001). For men, whether one was exposed to no ACEs (unstandardized *b* = 0.029 [SE = 0.016], *p* = 0.069) or 3+ ACEs (unstandardized *b* = −0.046 [SE = 0.032], *p* = 0.156), the relationship between length of longest deployment and baseline psychological symptoms was not significant.

Significant effects existed between the interaction of length of deployment x ACEs (1−2) and baseline psychological symptoms (unstandardized *b* = −0.107 [SE = 0.029], *p* < 0.001), slope (unstandardized *b* = 0.037 [SE = 0.017], *p* = 0.028) and quadratic (unstandardized *b* = −0.008 [SE = 0.003], *p* = 0.013) factors. Splitting the sample on ACEs and examining the effects of length of deployment on the intercept, slope, and quadratic factors yielded significant results. Among men who experienced 1−2 ACEs, those with a longer maximum length of deployment reported fewer mental health problems at baseline (unstandardized *b* = −0.047 [SE = 0.023], *p* = 0.037). In contrast, the relationship between the length of deployment and baseline psychological symptoms was non‐significant for men who reported 0 ACEs. Men with 1−2 ACEs and a longer maximum length of deployment had a steeper positive slope for psychological symptoms between Waves 1 and 2 (unstandardized *b* = 0.031 [SE = 0.015], *p* = 0.040). At the same time, this relationship was non‐significant for men with 0 ACEs. Finally, men with 1−2 ACEs and a longer maximum length of deployment had a significant inverse *U*‐shape in their longitudinal trend in psychological symptoms (unstandardized *b* = −0.007 [SE = 0.003], *p* = 0.021).

Regressing the intercept latent factor on the interaction of maximum length of deployment x combat exposure yielded significant results (unstandardized *b* = 0.124 [SE = 0.025], *p* < 0.001). Splitting the sample by combat exposure indicated that, among men who had one or more combat exposures, those with a longer maximum length of deployment reported significantly more psychological symptoms (unstandardized *b* = 0.088 [SE = 0.018], *p* < 0.001), compared to men without combat exposure (unstandardized *b* = −0.030 [SE = 0.017], *p* = 0.082).

### 3.4 Analyses of Interaction Effects Among Female Veterans

For women, the intercept latent factor regressed on the interaction between maximum length of deployment and combat exposure was significant (unstandardized *b* = 0.185 [SE = 0.054], *p* = 0.001). When the sample was split by combat exposure into 0 versus 1 or more exposures, women who reported no combat exposures had a non‐significant relationship between psychological symptoms at baseline and length of deployment (unstandardized *b* = −0.034 [SE = 0.034], *p* = 0.309), while women who reported at least 1 combat exposure had a significant association between psychological symptoms at baseline and length of deployment (unstandardized *b* = 0.136 [SE = 0.030], *p* < 0.001). Therefore, women who had combat exposure and a longer maximum length of deployment reported significantly greater mental health symptoms at baseline.

The regression of the slope factor on the interaction between length of deployment and combat exposure was also significant (unstandardized *b* = −0.087 [SE = 0.035], *p* = 0.001). When the sample was split by combat exposure, women who reported no exposure reported significant effects on the slope (unstandardized *b* = 0.053 [SE = 0.230], *p* = 0.024). At the same time, this relationship was non‐significant for women who reported one or more combat exposures (unstandardized *b* = −0.015 [SE = 0.023], *p* = 0.526). Therefore, women without combat exposure who had longer deployments evidenced a steeper positive slope, which indicates they reported more psychological symptoms over time.

## 4 Discussion

The current longitudinal study is one of the first to examine the unfolding of psychological symptoms as post‐9/11 veterans transition from military to civilian life. Few studies use longitudinal designs to examine the transitions of post‐9/11 veterans (Park et al. 2021). Previous research tended to use cross‐sectional data to make inferences regarding baseline levels of mental health problems or has used a limited 1‐year follow‐up period—also called the “critical year” (Park et al. 2021). In contrast, the current study included six waves of data which enables the examination of more nuanced results, including non‐linear relationships captured by the quadratic latent factor. The results revealed that the relationships between combat exposure and exposure to ACEs and psychological symptoms are complex; therefore, they are often not linear, change over time, and differ between male and female veterans.

When examining the entire sample, psychological symptom reports remained relatively flat over the first 3 years of the MCT. This was evidenced by non‐significant slope and quadratic estimates for both men and women. This finding contrasts with previous research that found modest longitudinal reductions in PTSD, anxiety, and insomnia symptoms (Park et al. 2021) and slightly increasing levels of depression and PTSD symptoms during the first year post‐discharge (Park et al. 2021). The addition of covariates in this study's analytical model and the subsequent identification of subgroups through interaction terms did reveal, however, significant linear and curvilinear trends in psychological symptoms.

Interestingly, this study's results indicate that the length of deployments is a significantly stronger predictor of psychological symptoms over time compared to the number of unique deployments that veterans experienced. The longer the deployment, the more negative impacts related to mental health. This result was consistent when examining the main effects and interactions. Long deployments separate service members from family, friends, and communities. During the global war on terror, service members, especially those in the Army, were commonly deployed for 12−18 months or longer. Service members may endure challenging conditions during longer deployments and face various stressors, including long work days, uncertain safety conditions, and often boredom (Bartone et al. 2008). Longer tours of duty are associated with decreased morale and mental health and disrupted relationships (Fear et al. 2010). In a review of the literature on the impact of deployment duration on health and well‐being, seven of the nine studies reviewed found duration was associated with poorer health outcomes (Buckman et al. 2011). Length of deployment among active‐duty service members is also associated with negative family outcomes, such as divorce and child problems in school (National Academy of Sciences 2019). Future studies of deployment length should include real‐time in‐theater measurement of deployment experiences and resulting service member and family member well‐being (Gromatsky et al. 2020).

The number of deployments was associated with lower psychological symptoms at baseline among both men and women; this outcome may suggest a steeling effect. For male post‐9/11 veterans, the number of deployments was also associated with decreasing psychological symptoms over time. Other studies have found that the number of deployments is not always related to psychological symptoms (Buckman et al. 2011; Fear et al. 2010). Indeed, a high proportion of service members see positives from deploying. For example, service members earn more money, make stronger connections to peers, and enhance their opportunities for promotion (Newby et al. 2005). Perhaps more frequent, but shorter deployments (i.e., 12 months or less), are healthier for service members and shorter deployments may help build coping skills and emotional resilience while also allowing more frequent breaks to reconnect with family and friends (Rona et al. 2007). In addition, short deployments may mean less disruption to the generally predictable patterns in life. Why these positive mental health effects associated with more deployments increased for males over time but not for females is perplexing. Future research should attempt to disentangle how male and female veterans are impacted by specific characteristics of deployments.

Childhood trauma and combat exposure are two types of stressors that can negatively affect the well‐being veterans (Doucette et al. 2023; Morgan et al. 2021). The deleterious effects of experiencing these stressors on mental health has been well established in cross‐sectional studies (Nichter et al. 2020). The relationship between childhood trauma and mental health has also been well established in military populations (Institute of Medicine 2013). The results from this study support these prior findings as at baseline, among men and women, veterans with ACEs scores of 1−2 reported significantly more psychological symptoms than their counterparts who reported no ACEs. In addition, male and female veterans with three or more ACEs reported significantly more psychological symptoms than veterans who reported 1−2 ACEs. Men who reported 1−2 ACEs, compared to men with none, experienced significantly steeper upward slopes, which indicates they had increasing psychological symptoms over time. However, slopes were non‐significant and, therefore, flat for women who reported 1‐2 and 3 or more ACEs, which indicates that women were initially above average at baseline and remained so during the study. The steeper positive slope for men suggests declining mental health may be due, at least in part, to help‐seeking stigma and barriers to care (RAND 2014) and lack of social connection (Wilson et al. 2018).

In terms of combat exposure effects on mental health, results from this study only partially support previous findings of the negative relationship between combat exposure and mental health. At baseline, men and women who reported combat exposure, regardless of the level of exposure, had elevated levels of psychological symptoms compared to their counterparts who did not have combat exposure. In addition, women, as compared to men, had steeper negative slopes, which indicates their mental health problems subsided for a short period (i.e., from Waves 1−2), but the significant positive effect on the quadratic factor indicates a rebounding effect on reported mental health problems for women. These nuanced effects of combat exposure on the slope and quadratic factors were significantly different between men and women, which indicates a flat (non‐significant) slope for men that was elevated at baseline and remained so for the duration of the study. This significant difference in slopes may be due to women tending to pursue more outpatient mental health services that may help them better cope with and reduce their problematic mental health symptoms (Kimerling et al. 2015).

### 4.1 Limitations

While the present data set represents one of the largest and most comprehensive to date, it also had differential attrition as is the case in most longitudinal studies. However, to remedy the potential biases of attrition, the researchers included covariates known to be associated with missing data in studies of veterans using auxiliary variables in the latent growth curve models (Vogt et al. 2018). In addition, the mental health problem variables were self‐reported even though the survey items came from validated questionnaires, including the PHQ‐4 and the PHQ‐9. Although latent growth curve modeling imposes a relatively strict functional form on the data, the excellent fit indices, used in this study, indicate that imposing intercept, slope, and quadratic factors on the data produced a well‐fitting model that could be used to study relationships among predictors and outcomes in the data. Nonetheless, the use of brief screeners provides only a glimpse of the respondent's experience and in no way indicates the presence of a clinically significant problem. Reports of ACEs required significant retrospection and, therefore, may be impacted by recall bias. The prevalence of ACEs exposure was lower in this study than in others. Rates are typically higher when those who use VA mental health clinics and veterans of all wars are sampled. Finally, the sample was not random, nor was it drawn from a clinical population of veterans. Both factors could reduce the generalizability of the conclusions.

## 5 Conclusion

Identifying and recognizing temporal influences on MCTs are important. The current study demonstrated that pre‐military and during‐military factors are variably associated with veteran psychological symptoms. Using mental health screeners over time, mental health providers could better track changes veteran well‐being and adjust interventions accordingly. Mental health providers also need to be sensitive to potential differences in how males and females respond to combat and other traumatic experiences. Lastly, this study also suggests that the length of deployment may be more predictive of psychological symptoms than the number of unique deployments. The Department of Defense might consider strategies to reduce the length of deployments in the future.

## Ethics Statement

Ethical approval for this study was obtained from: ICF International Inc. Protocol Number: 151636.0.000.00.000. Study Title: “The Veterans Metrics Initiative: Linking Program Components to Post‐Military Well‐Being.” Approval Date: March 19, 2019.

## Conflicts of Interest

The authors declare no conflicts of interest.

## Data Availability

The data that support the findings of this study are openly available in ICPSR at https://www.icpsr.umich.edu/web/ICPSR/studies/38051, reference number ICPSR 38051.

## References

[jclp70038-bib-0001] Ahern, J. , M. Worthen , J. Masters , S. A. Lippman , E. J. Ozer , and R. Moos . 2015. “The Challenges of Afghanistan and Iraq Veterans' Transition From Military to Civilian Life and Approaches to Reconnection.” PLoS One 10, no. 7: e0128599. 10.1371/journal.pone.0128599. 26132291 PMC4489090

[jclp70038-bib-0002] Anda, R. F. , V. J. Felitti , J. D. Bremner , et al. 2006. “The Enduring Effects of Abuse and Related Adverse Experiences in Childhood.” European Archives of Psychiatry and Clinical Neuroscience 256, no. 3: 174–186. 10.1007/s00406-005-0624-4. 16311898 PMC3232061

[jclp70038-bib-0003] Aronson, K. R. , D. F. Perkins , N. R. Morgan , et al. 2020. “The Impact of Adverse Childhood Experiences (ACEs) and Combat Exposure on Mental Health Conditions Among New Post‐9/11 Veterans.” Psychological Trauma: Theory, Research, Practice, and Policy 12, no. 7: 698–706. 10.1037/tra0000614. 32614200

[jclp70038-bib-0004] Bartone, P. T. , R. R. Roland , J. J. Picano , and T. J. Williams . 2008. “Psychological Hardiness Predicts Success in US Army Special Forces Candidates.” International Journal of Selection and Assessment 16, no. 1: 78–81. 10.1111/j.1468-2389.2008.00412.x.

[jclp70038-bib-0005] Blosnich, J. R. , M. E. Dichter , C. Cerulli , S. V. Batten , and R. M. Bossarte . 2014. “Disparities in Adverse Childhood Experiences Among Individuals With a History of Military Service.” JAMA Psychiatry 71, no. 9: 1041–1048. 10.1001/jamapsychiatry.2014.724. 25054690 PMC8981217

[jclp70038-bib-0006] Bokhour, B. G. , J. N. Haun , J. Hyde , M. Charns , and B. Kligler . 2020. “Transforming the Veterans Affairs to a Whole Health System of Care: Time for Action and Research.” Medical Care 58, no. 4: 295–300. 10.1097/MLR.0000000000001316. 32044866

[jclp70038-bib-0007] Buckman, J. E. J. , J. Sundin , T. Greene , et al. 2011. “The Impact of Deployment Length on the Health and Well‐Being of Military Personnel: A Systematic Review of the Literature.” Occupational and Environmental Medicine 68, no. 1: 69–76. 10.1136/oem.2009.054692. 20884791

[jclp70038-bib-0008] Campbell, M. S. , K. O'Gallagher , D. J. Smolenski , et al. 2021. “Longitudinal Relationship of Combat Exposure With Mental Health Diagnoses in the Military Health System.” Military Medicine 186, no. S1: 160–166. https://academic.oup.com/milmed/article/186/Supplement_1/160/6119456. 33499480 10.1093/milmed/usaa301

[jclp70038-bib-0009] Chesnut, R. P. , C. B. Richardson , N. R. Morgan , et al. 2020. “Moral Injury and Social Well‐Being: A Growth Curve Analysis.” Journal of Traumatic Stress 33, no. 4: 587–597. 10.1002/jts.22567. 32662166

[jclp70038-bib-0010] Cramm, H. , D. Norris , K. D. Schwartz , L. Tam‐Seto , A. Williams , and A. Mahar . 2020. “Impact of Canadian Armed Forces Veterans' Mental Health Problems on the Family During the Military to Civilian Transition.” Military Behavioral Health 8, no. 2: 148–158. 10.1080/21635781.2019.1644260.

[jclp70038-bib-0011] Curran, P. J. 2000. “A Latent Curve Framework for the Study of Developmental Trajectories in Adolescent Substance Use.” In Multivariate Applications in Substance Use Research, Edited by J. S. Rose , L. Chassin , C. C. Presson , and S. J. Sherman , 1–42. Psychology Press.

[jclp70038-bib-0012] Derefinko, K. J. , T. A. Hallsell , M. B. Isaacs , L. W. Colvin , F. I. Salgado Garcia , and Z. Bursac . 2019. “Perceived Needs of Veterans Transitioning from the Military to Civilian Life.” Journal of Behavioral Health Services & Research 46, no. 3: 384–398. 10.1007/s11414-018-9633-8. 30218429

[jclp70038-bib-0013] Doucette, C. E. , N. R. Morgan , K. R. Aronson , J. A. Bleser , K. J. McCarthy , and D. F. Perkins . 2023. “The Effects of Adverse Childhood Experiences and Warfare Exposure on Military Sexual Trauma Among Veterans.” Journal of Interpersonal Violence 38: 3777–3805. https://pubmed.ncbi.nlm.nih.gov/35962589/. 35962589 10.1177/08862605221109494 PMC9850385

[jclp70038-bib-0014] Fear, N. T. , M. Jones , D. Murphy , et al. 2010. “What Are the Consequences of Deployment to Iraq and Afghanistan on the Mental Health of the UK Armed Forces? A Cohort Study.” Lancet 375, no. 9728: 1783–1797. 10.1016/S0140-6736(10)60672-1. 20471076

[jclp70038-bib-0015] Felitti, V. J. , R. F. Anda , D. Nordenberg , and D. F. Williamson . 1998. “Adverse Childhood Experiences and Health Outcomes in Adults: The ACE Study.” Journal of Family and Consumer Sciences 90, no. 3: 31. https://www.proquest.com/scholarly-journals/adverse-childhood-experiences-health-outcomes/docview/218184173/se-2.

[jclp70038-bib-0016] Galovski, T. , and J. A. Lyons . 2004. “Psychological Sequelae of Combat Violence: A Review of the Impact of PTSD on the Veteran's Family and Possible Interventions.” Aggression and Violent Behavior 9, no. 5: 477–501. 10.1016/S1359-1789(03)00045-4.

[jclp70038-bib-0017] Gaylord, K. M. 2006. “The Psychosocial Effects of Combat: The Frequently Unseen Injury.” Critical Care Nursing Clinics of North America 18, no. 3: 349–357. 10.1016/j.ccell.2006.05.010. 16962456

[jclp70038-bib-0018] Gromatsky, M. , S. R. Sullivan , A. P. Spears , et al. 2020. “Ecological Momentary Assessment (EMA) of Mental Health Outcomes in Veterans and Servicemembers: A Scoping Review.” Psychiatry Research 292: 113359. 10.1016/j.psychres.2020.113359. 32777594

[jclp70038-bib-0019] Hu, L. , and P. M. Bentler . 1999. “Cutoff Criteria for Fit Indexes in Covariance Structure Analysis: Conventional Criteria Versus New Alternatives.” Structural Equation Modeling: A Multidisciplinary Journal 6, no. 1: 1–55. 10.1080/10705519909540118.

[jclp70038-bib-0020] Inoue, C. , E. Shawler , C. H. Jordan , M. J. Moore , and C. A. Jackson . 2021. Veteran and Military Mental Health Issues. StatPearls Publishing. https://europepmc.org/article/NBK/nbk572092. 34283458

[jclp70038-bib-0021] Institute of Medicine . 2010. Returning Home from Iraq and Afghanistan: Preliminary Assessment of Readjustment Needs of Veterans, Service Members, and Their Families. The National Academy of Sciences. 25032369

[jclp70038-bib-0022] Institute of Medicine . 2013. Returning Home from Iraq and Afghanistan: Assessment of Readjustment Needs of Veterans, Service Members, and Their Families. The National Academy of Sciences. 24901192

[jclp70038-bib-0023] Joseph, J. S. , L. Smith‐MacDonald , M. C. Filice , and M. S. Smith . 2023. “Reculturation: A New Perspective on Military‐Civilian Transition Stress.” Military Psychology 35, no. 3: 193–203. 10.1080/08995605.2022.2094175. 37133548 PMC10198009

[jclp70038-bib-0024] Karre, J. K. , D. F. Perkins , N. R. Morgan , et al. 2024. “What Do Successful Military‐to‐Civilian Transitions Look Like? A Revised Framework and a New Conceptual Model for Assessing Veteran Well‐Being.” Armed Forces & Society 51, no. 3: 1–30. 10.1177/0095327X231216678.

[jclp70038-bib-0025] Kimerling, R. , L. A. Bastian , B. A. Bean‐Mayberry , et al. 2015. “Patient‐Centered Mental Health Care for Female Veterans.” Psychiatric Services 66, no. 2: 155–162. 10.1176/appi.ps.201300551. 25642611 PMC4776740

[jclp70038-bib-0026] King, D. , L. King , and D. S. Vogt . 2003. Manual for the Deployment Risk and Resilience Inventory (DRRI): A Collection of Measures for Studying Deployment‐Related Experiences of Military Veterans. National Center for PTSD.

[jclp70038-bib-0027] Kroenke, K. , R. L. Spitzer , J. B. Williams , and B. Löwe . 2009. “An Ultra‐Brief Screening Scale for Anxiety and Depression: The PHQ–4.” Psychosomatics 50, no. 6: 613–621. 10.1016/s0033-3182(09)70864-3. 19996233

[jclp70038-bib-0028] Laird, C. W. , and P. Alexander . 2019. “Prevalence of Adverse Childhood Experiences Among Veterans.” Clinical Social Work Journal 47, no. 4: 384–393. 10.1007/s10615-019-00703-5.

[jclp70038-bib-0029] LeardMann, C. A. , B. Smith , and M. A. Ryan . 2010. “Do Adverse Childhood Experiences Increase the Risk of Postdeployment Posttraumatic Stress Disorder in US Marines?” BMC Public Health 10, no. 1: 437. 10.1186/1471-2458-10-437. 20659342 PMC2916906

[jclp70038-bib-0030] Milanak, M. E. , D. F. Gros , K. M. Magruder , O. Brawman‐Mintzer , and B. C. Frueh . 2013. “Prevalence and Features of Generalized Anxiety Disorder in Department of Veteran Affairs Primary Care Settings.” Psychiatry Research 209, no. 2: 173–179. 10.1016/j.psychres.2013.03.031. 23659756 PMC4026032

[jclp70038-bib-0031] Morgan, N. R. , K. R. Aronson , D. F. Perkins , et al. 2021. “The Interaction of Exposure to Adverse Childhood and Combat Experiences on the Current Mental Health of New Post‐9/11 Veterans.” Journal of Community Psychology 50: 204–220. 33624843 10.1002/jcop.22523

[jclp70038-bib-0032] Morin, R. 2011. The Difficult Transition from Military to Civilian Life. Pew Research Center.

[jclp70038-bib-0033] Muthen, L. K. , and B. O. Muthen . 2017. MPlus: Statistical Analysis With Latent Variables User's Guide. Muthen & Muthen.

[jclp70038-bib-0034] National Academy of Sciences . 2019. Strengthening the Military Family Readiness System for a Changing American Society. National Academy of Sciences. 31600043

[jclp70038-bib-0035] Newby, J. H. , J. E. McCarroll , R. J. Ursano , Z. Fan , J. Shigemura , and Y. Tucker‐Harris . 2005. “Positive and Negative Consequences of a Military Deployment.” Military Medicine 170, no. 10: 815–819. 10.7205/MILMED.170.10.815. 16435750

[jclp70038-bib-0036] Nichter, B. , M. Hill , S. Norman , M. Haller , and R. H. Pietrzak . 2020. “Associations of Childhood Abuse and Combat Exposure With Suicidal Ideation and Suicide Attempt in US Military Veterans: A Nationally Representative Study.” Journal of Affective Disorders 276: 1102–1108. 10.1016/j.jad.2020.07.120. 32777648

[jclp70038-bib-0037] Norman, S. B. , M. Haller , J. L. Hamblen , S. M. Southwick , and R. H. Pietrzak . 2018. “The Burden of Co‐Occurring Alcohol Use Disorder and PTSD in US Military Veterans: Comorbidities, Functioning, and Suicidality.” Psychology of Addictive Behaviors 32, no. 2: 224–229. 10.1037/adb0000348. 29553778

[jclp70038-bib-0038] Park, C. L. , S. J. Sacco , L. Finkelstein‐Fox , et al. 2021. “Post‐9/11 Military Veterans' Adjustment to Civilian Life Over Time Following Separation From Service.” Journal of Clinical Psychology 77, no. 9: 2077–2095. 10.1002/jclp.23144. 33871869

[jclp70038-bib-0039] Pedlar, D. , J. M. Thompson , and C. A. Castro . 2019. “Military‐to‐Civilian Transition Theories and Frameworks.” In Military Veteran Reintegration, Edited by D. Pedlar , J. M. Thompson , and C. A. Castro , 21–50. Elsevier Academic Press.

[jclp70038-bib-0040] Perkins, D. F. , K. R. Aronson , N. R. Morgan , et al. 2020. “Veterans’ Use of Programs and Services as They Transition to Civilian Life: Baseline Assessment for the Veteran Metrics Initiative.” Journal of Social Service Research 46, no. 2: 241–255. 10.1080/01488376.2018.1546259.

[jclp70038-bib-0041] RAND . 2014. Mental Health Stigma in the Military. RAND Corporation.

[jclp70038-bib-0042] Rona, R. J. , N. T. Fear , L. Hull , et al. 2007. “Mental Health Consequences of Overstretch in the UK Armed Forces: First Phase of a Cohort Study.” BMJ 335, no. 7620: 603. 10.1136/bmj.39274.585752.BE. 17664192 PMC1988977

[jclp70038-bib-0043] Sayer, N. A. , K. F. Carlson , and P. A. Frazier . 2014. “Reintegration Challenges in US Service Members and Veterans Following Combat Deployment.” Social Issues and Policy Review 8, no. 1: 33–73. 10.1111/sipr.12001.

[jclp70038-bib-0045] Sher, L. , M. D. Braquehais , and M. Casas . 2012. “Posttraumatic Stress Disorder, Depression, and Suicide in Veterans.” Cleveland Clinic Journal of Medicine 79, no. 2: 92–97. 10.3949/ccjm.79a.11069. 22301558

[jclp70038-bib-0046] US Army Surgeon General . 2005. Operation lraqi Freedom (01F‐11) Mental Health Advisory Team (MHAT‐II) Report. Department of the Army. https://www.govinfo.gov/content/pkg/GOVPUB-D104-PURL-LPS62196/pdf/GOVPUB-D104-PURL-LPS62196.pdf.

[jclp70038-bib-0047] U.S. Bureau of Labor Statistics . 2020. Employment Situation of Veterans—2019. U.S. Department of Labor.

[jclp70038-bib-0048] Vogt, D. , D. F. Perkins , L. A. Copeland , et al. 2018. “The Veterans Metrics Initiative Study of US Veterans' Experiences During Their Transition from Military Service.” BMJ Open 8, no. 6: e020734. 10.1136/bmjopen-2017-020734 PMC6009506 29895650

[jclp70038-bib-0049] Vogt, D. , B. N. Smith , L. A. King , D. W. King , J. Knight , and J. J. Vasterling . 2013. “Deployment Risk and Resilience Inventory‐2 (DRRI‐2): An Updated Tool for Assessing Psychosocial Risk and Resilience Factors Among Service Members and Veterans.” Journal of Traumatic Stress 26, no. 6: 710–717. 10.1002/jts.21868. 24490250

[jclp70038-bib-0050] Wilson, G. , M. Hill , and M. D. Kiernan . 2018. “Loneliness and Social Isolation of Military Veterans: Systematic Narrative Review.” Occupational Medicine 68, no. 9: 600–609. 10.1093/occmed/kqy160. 30551163

